# ST-Segment Depression in Hyperventilation Indicates a False Positive Exercise Test in Patients with Mitral Valve Prolapse

**DOI:** 10.4061/2010/541781

**Published:** 2010-11-14

**Authors:** Andreas P. Michaelides, Charalampos I. Liakos, Charalambos Antoniades, Dimitrios L. Tsiachris, Dimitrios Soulis, Polichronis E. Dilaveris, Konstantinos P. Tsioufis, Christodoulos I. Stefanadis

**Affiliations:** ^1^Exercise Laboratory, 1st University Department of Cardiology, Athens Medical School, Hippokration Hospital, 15772 Athens, Greece; ^2^Catheterization Laboratory, 1st University Department of Cardiology, Athens Medical School, Hippokration Hospital, 15772 Athens, Greece

## Abstract

*Objectives*. Mitral valve prolapse (MVP) is a known cause for false positive exercise test (ET). The purpose of this study was to establish additional electrocardiographic criteria to distinguish the false positive exercise results in patients with MVP. *Methods*. We studied 218 consecutive patients (53 ± 6 years, 103 males) with MVP (according to echocardiographic study), and positive treadmill ET was performed due to multiple cardiovascular risk factors or angina-like symptoms. A coronary angiography was performed to detect coronary artery disease (CAD). *Results*. From 218 patients, 90 (group A) presented with normal coronary arteries according to the angiography (false positive ET) while the rest 128 (group B) presented with CAD. ST-segment depression in hyperventilation phase was present in 54 patients of group A (60%) while only in 14 patients of group B (11%), *P* < .05. *Conclusions*. Presence of ST-segment depression in hyperventilation phase favors a false positive ET in patients with MVP.

## 1. Introduction

Mitral valve prolapse (MVP) is one of the most prevalent cardiac valvular abnormalities. Using standardized echocardiographic diagnostic criteria, a community-based study showed that MVP occurs in 2.4 percent of the population [1, 2]. It is twice as frequent in females as in males. The clinical and echocardiographic criteria for the diagnosis of MVP have been well established. The characteristic systolic click and mid-to-late systolic murmur is a major diagnostic criterion. The most specific echocardiographic criterion is superior displacement of one or both mitral valve leaflets by more than 2 mm above the plane of the annulus [2, 3] in the long axis.

 The vast majority of patients with MVP are asymptomatic and remain so throughout their lives. However, some patients may complain of symptoms like syncope, pre-syncope, palpitations, chest discomfort, and, when mitral regurgitation (MR) is severe, symptoms of diminished cardiac reserve. Chest discomfort may be typical of angina pectoris but is more often atypical in that it is prolonged, not clearly related to exertion, and punctuated by brief attacks or severe stabbing pain at the apex.

 In these patients false positive exercise stress responses, as testified by coronary angiography, electrocardiographic [4–14] as well as with thallium perfusion imaging [12, 15, 16] both have been consistently reported in several studies.

 Since the diagnosis of coronary artery disease (CAD) in patients with MVP based on the standard ST-segment depression criteria during exercise testing (ET) is uncertain, the establishment of accessional electrocardiographic (ECG) criteria for the discrimination of true positive exercise responses would be of great value. 

 It is well known that patients with MVP often have high levels of catecholamines [17–22]. On the other hand, patients with high levels of catecholamines frequently present with ST-segment depression during hyperventilation in the absence of CAD [23–27]. We examined whether the presence of ST-segment depression during hyperventilation in patients with MVP leads to false positive ETs.

## 2. Materials and Methods

### 2.1. Study Population

From 2006 to 2009, 218 consecutive patients with MVP (103 males, 58 ± 8 years) and a normal resting ECG who presented with ischemic ECG changes during ET and agreed to undergo coronary angiography were included in the study. During the recruitment period, a total of 746 patients with MVP were examined. From them, 252 (34%) presented with ischemic ECG changes during ET in our laboratory, and 218 (87%) accepted to participate in our study.

 The diagnosis of MVP was based on an echocardiographic study while the definition of ischemic ECG response during ET was based on the standard ST-segment deviation criteria. Treadmill ET was performed in these patients due to multiple cardiovascular risk factors or angina-like symptoms.

 Patients with known history of CAD, left or right bundle-branch block, left or right ventricular hypertrophy, other valvular abnormality, pre-excitation syndromes, and those who were receiving digitalis or were unable to walk on treadmill were excluded from the study.

 All medications were discontinued for at least 5 half-lives before the ET.

 The study was approved by our hospital's committee on human research, and written informed consent was obtained from all participants.

### 2.2. Echocardiographic Study

Echocardiographic study was performed to all patients in order to establish the diagnosis of MVP [16]. The two-dimensional echocardiogram showed that one or both mitral valve leaflets billow by at least 2 mm into the left atrium during systole in the long-axis view [3, 28, 29]. Thickening of the involved leaflet to greater than 5 mm was often observed supporting the diagnosis. Findings like increased leaflet area, leaflet redundancy, chordal elongation, and annular dilation were indicative of more severe myxomatous disease.

 From 218 patients with documented prolapse, 180 (83%) had typical auscultatory findings (click or murmur).

 Doppler echocardiography frequently revealed mild MR that was not always associated with an audible murmur. Moderate to severe MR was present in about two-thirds of patients with posterior leaflet prolapse and in about one-fourth of patients with anterior leaflet prolapse which is in concordance with the literature [30].

### 2.3. Treadmill ET—Hyperventilation

All patients performed ET on a Marquette case system (GE-Medical System, Milwaukee, WI), according to the multistage Bruce protocol.

 Each patient had an electrocardiogram (ECG) recorded with the standard 12 leads. The ECG was recorded continuously at the resting status (both supine and standing), during hyperventilation, during exercise, and for up to the 10th minute of the recovery period. Blood pressure was measured every minute at the resting status (both supine and standing), during hyperventilation, during exercise, and during the recovery period, with a sphygmomanometer.

 Exercise was terminated in the presence of exercise-limiting angina, fatigue, dyspnea, ataxia, dizziness, near syncope, or signs of poor perfusion (cyanosis or pallor). In the absence of symptoms, the test was terminated at the occurrence of ≥ 0.2 mV ST-segment depression or ≥ 0.1 mV ST-segment elevation, an exaggerated blood pressure response (≥ 250/115 mm Hg), a decrease in systolic blood pressure ≥ 10 mm Hg from baseline or specific arrhythmias (sustained ventricular tachycardia, multifocal ventricular premature beats, ventricular triplets, supraventricular tachycardia, heart block, or bradyarrhythmia) [31].

 All patients underwent forced hyperventilation (breaths as deep and rapid as possible—at least 30 breaths/min) in the standing position before the exercise. The test required the subject to overbreadth voluntarily the room air (FiO_2_: 21%) with a frequency rate of 40–45 breaths/min at room temperature (20–22°C). The target ventilation rate was equivalent to the maximum voluntary ventilation, and the mean duration of the test was 1 minute. Hyperventilation was discontinued earlier if the patient felt dizziness, lightheadedness, weakness, shortness of breath, a sense of unsteadiness, tingling around the mouth, fingertips, and toes (as a result of hypocapnia) in order to avoid loss of consciousness.

 The interpretation of exercise testing included symptomatic response, exercise duration, blood pressure, heart rate, and ECG response.

 An ischemic ST-segment response during exercise and recovery was defined (according to the standard ST-segment deviation criteria) as horizontal or downsloping depression of ≥ 0.1 mV below the resting ST-segment level (measured 60 milliseconds after the J-point), upsloping depression of ≥ 0.15 mV (measured 80 milliseconds after J-point), or elevation of ≥ 0.1 mV [31].

**Figure 1:**
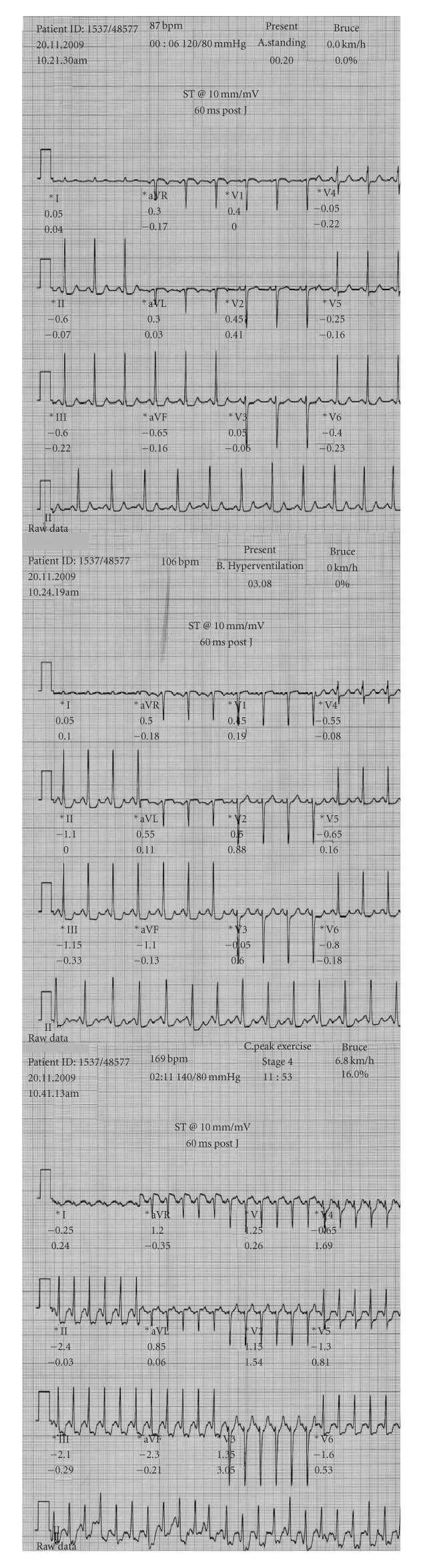
Electrocardiographic (ECG) recordings from a 48-year old woman with mitral valve prolapse undergoing maximal treadmill test. A. Resting status-standing position-normal breathing: normal ECG, B. Resting status-standing position-hyperventilation: ST-segment depression in leads II, III, aVF (*≈* 0.13 mV)/V_4_, V_5_, V_6_ (*≈* 0.1 mV), C. Peak exercise-standing position: ST-segment depression in leads II, III, aVF (*≈* 0.23 mV)/V_4_, V_5_, V_6_ (*≈* 0.16 mV). This was a false positive exercise test since this woman had a normal coronary arteriography.

 Intensive care was taken to detect ST-segment depression in any of the leads during the hyperventilation phase of the test since all patients presented with normal baseline (both supine and standing) ECG. In the presence of ST-segment depression during hyperventilation, an ischemic ST-segment response during exercise was defined as additional depression of ≥ 0.1 mV (measured in the standing position) below the hyperventilation ST-segment level (Figure 1).

 Exercise tests without ischemic ST-segment changes, which were terminated when the heart rate was < 85% of the predicted maximal heart rate, were considered inconclusive. Patients with inconclusive exercise tests were excluded from the study.

 ECG measurements were performed with a magnifying lens by two of the investigators who were unaware of the results of the coronary angiography. The interobserver and intraobserver variability for ST-segment changes was 0.007 ± 0.005 and 0.008 ± 0.004 mV, respectively. Disagreements between observers were resolved by a third interpreter.

### 2.4. Coronary Arteriography

Selective coronary arteriography was performed to all patients, using the percutaneous (Judkins) technique. The left coronary artery was visualized at the 60° left anterior oblique, the 30° right anterior oblique, and the left lateral position, with a 30° cranial angulation. The right coronary artery was visualized at the 60° left anterior oblique and the 30° right anterior oblique position.

 Significant coronary stenosis was defined as diameter narrowing of 70% or more in the lumen of left anterior descending (LAD), left circumflex (LCx), and right coronary artery (RCA), or 50% or more in the lumen of left main coronary artery. The interpretation was performed by two investigators who were unaware of ET results.

### 2.5. Statistical Analysis

All continuous variables were normally distributed as confirmed by using Kolmogorov-Smirnov test and are expressed as mean ± SD. Categorical variables are reported as observed number (percentage). Comparisons of continuous variables between two groups were performed by using unpaired *t*-test. Categorical variables between two groups were compared using chi-square test (with the use of Yates correction) or Fisher's exact *t*-test. All tests were two-sided. Differences were considered as statistically significant for *P*  value < .05. Data analysis was performed with SPSS 15.0 statistical software (SPSS Inc., Chicago, IL, USA).

## 3. Results

From 218 patients included in the study, 103 (47%) were males while the rest 115 (53%) patients were females.

 Patient's characteristics, echocardiographic and angiographic data are demonstrated in Table 1.

 There were no differences between males/females regarding the mean age of the patients or the prevalence of arterial hypertension, diabetes mellitus, hypercholesterolemia, and smoking.

 Anterior leaflet prolapse was present in 80 males (78%) and 77 females (67%), *P* = .048 while posterior leaflet prolapse was present in 23 males (22%) and 38 females (33%), *P* = .042. The ejection fraction of the left ventricle was similar in men and women.

 Normal coronary arteries were found in 38 males (37%) and 52 females (45%) while 1-vessel disease was found in 33 males (32%) and 37 females (32%), 2-vessel disease in 21 males (20%) and 18 females (16%), and 3-vessel disease in 11 males (11%) and 8 females (7%), *P* = NS for all. Overall, from 218 patients with positive ET, 90 (41%) had normal coronary angiograms (false positive ETs = 41%). Coronary vasospasm was not observed in any patient during angiography.

 Exercise and ECG data are presented in Table 2.

 Exercise parameters (resting/hyperventilation/maximal systolic blood pressure, resting/hyperventilation/maximal heart rate, percentage of exercise-induced angina) were similar in males and females with the exception of the duration (in sec) of the exercise (520 ± 32 for males versus 470 ± 25 for females, *P* = .036). Hyperventilation induced an increase in systolic blood pressure (8 ± 3 versus 8 ± 4 mmHg for males and females, resp., *P* = NS) and heart rate (26 ± 4 versus 28 ± 4 bpm for males and females, resp., *P* = NS).

 ST-segment depression during the hyperventilation phase of the test was detected in 27 males (26%) and 41 females (36%), *P* = .041. From them, 31 patients (46%) presented with ST-segment depression in the inferior leads (II, III, aVF), 2 patients (3%) in the anterior leads (V_1_, V_2_, V_3_), 12 patients (18%) in the lateral leads (V_4_, V_5_, V_6_) while the rest 23 patients (34%) in both inferior and lateral leads. Furthermore, 27 patients (40%) presented with horizontal and the rest 41 patients (60%) with downsloping pattern of ST-segment depression. This depression was measured (in mV) up to 0.09 ± 0.03 in men versus 0.11 ± 0.05 in women, *P* = NS (0.09 ± 0.04 versus 0.11 ± 0.05 for the inferior leads, 0.02 ± 0.02 versus 0.01 ± 0.01 for the anterior leads, and 0.09 ± 0.03 versus 0.1 ± 0.01 for the lateral leads in men and women, resp., *P* = NS for all).

 Maximal exercise-induced ST-segment depression (in mV) was measured at peak exercise up to 0.15 ± 0.02 in men versus 0.19 ± 0.03 in women, *P* = NS (0.12 ± 0.05 versus 0.19 ± 0.03 for the inferior leads—*P* = .044—, 0.04 ± 0.03 versus 0.01 ± 0.01 for the anterior leads—*P* = NS—and 0.11 ± 0.02 versus 0.18 ± 0.01 for the lateral leads—*P* = .041— in men and women, resp.).

 Time required for exercise-induced ST-segment depression of 0.1 mV (in sec) was greater in males (390 ± 16 versus 362 ± 12,  *P* = .043).

**Figure 2:**
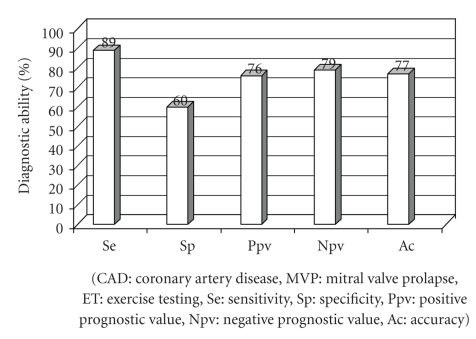
Diagnostic ability of a normal electrocardiogram during hyperventilation in detecting CAD in patients with MVP and positive ET.

 The correlation of ST-segment depression at hyperventilation with angiographic result is presented in Table 3. Patients with normal coronary arteries appeared with ST-segment depression during hyperventilation more frequently versus them with CAD (55% versus 9% for men, 63% versus 13% for women, 60% versus 11% for total population, *P* < .05 for all). Overall, from 68 patients with ST-segment depression during hyperventilation, 54 (79%) appeared without CAD (Table 3, Figure 1) since from 150 patients without ST-segment depression during hyperventilation, only 36 (24%) appeared without CAD (false positive ETs = 24%). According to these data, sensitivity (Se), specificity (Sp), positive prognostic value (Ppv), negative prognostic value (Npv), and diagnostic accuracy (Ac) of the absence of ST-segment depression during hyperventilation in detecting CAD in patients with MVP and positive ET were calculated (up to 89, 60, 76, 79, and 77%, resp.) and are presented in Figure 2.

## 4. Discussion

The prevalence of abnormal exercise tests in patients with MVP varies in the literature from 10 to 60% [4–14]; the overall prevalence, however, is in the region of 33% [11]. 

 The results of this study indicate that false positive exercise response is common in patients with MVP (*≈* 41%) which is concordant with the literature [4–16].

 Hyperventilation-induced ST-segment depression is a criterion that (according to the results of this study) makes CAD less likely in MVP patients with exercise-induced ischaemic response. The incorporation of this new electrocardiographic criterion in the standard ST-segment depression criteria makes exercise stress testing much more accurate in detecting CAD in MVP patients. With sensitivity up to 89% and reduced false positive responses compared to standard ET (from 41% to 24%), a normal ECG during hyperventilation makes an ischaemic stress response in patients with MVP quite trustworthy.

 No previous data are available concerning the ability of ST-segment changes during hyperventilation to detect coronary artery disease in patients with MVP when accompanying with positive exercise-induced responses. Ellestad et al. report that patients with abnormal autonomic drive (high levels of catecholamines) have demonstrated ST-segment depression after hyperventilation as well after exercise [23]. In their experience, patients who display ST-segment depression at rest or an increased ST-segment depression after hyperventilation, in whom the depression tends to return to normal with exercise, usually do not have epicardial CAD [23]. Jacobs et al. [24] also report that changes associated with hyperventilation are usually related with normal coronary arteries. Propranolol and other beta blockers have been shown to block the ST-segment changes associated with hyperventilation, suggesting an autonomic etiology [25–27]. 

 Mechanisms involving disturbed balance between oxygen demand and supply, decreased level of high-energy phosphate stores, changes in electrolyte contents, intracellular Ca^2+^ overload, and oxidative stress play essential role in the pathogenesis of catecholamine-induced myocardial damage [32]. Several studies have shown that under stressful conditions high concentrations of catecholamines become oxidized to form aminolutins and generate oxyradicals. These oxidation products of catecholamines have been demonstrated to produce coronary spasm, arrhythmias, and cardiac dysfunction by inducing Ca^2+^-handling abnormalities in both sarcolemmal and sarcoplasmic reticulum, defects in energy production by mitochondria, and myocardial cell damage [32]. Hyperventilation-induced ST-segment depression, blood pressure, and heart rate increase in patients with MVP is probably due to excessive amount of catecholamines, increased oxygen demand, and coronary spasm [17–22, 33] and may be prevented by beta-adrenergic blockers, which are useful for lowering oxygen consumption and downregulating sympathetic nervous system activity [27]. 

 Considering all these potential pathophysiological mechanisms and that patients with high levels of catecholamines frequently present with ST-segment depression during hyperventilation in the absence of CAD [25–27], the improvement in diagnostic ability of exercise testing in our study with the use of this novel ECG criterion seems reasonable.

## 5. Conclusions

Absence of ST-segment changes during hyperventilation in MVP patients with positive exercise testing is proved to be a useful diagnostic tool for detecting CAD with Se, Sp, Ppv, Npv, and Ac up to 89, 60, 76, 79, and 77%, respectively. Reducing false positive responses (from 41% to 24%), this novel criterion could guide physicians to avert unnecessary coronary arteriography in an amount of patients with MVP (especially them without angina-like symptoms during exercise). Since exercise ECG test is a popular, well-established, inexpensive, widely available procedure, we should probably consider it as an acceptable diagnostic tool for CAD even in patients with MVP after incorporating this novel ECG criterion.

## 6. Limitations

The results of our study would be more explicable if catecholamines levels had been measured in our patients. Besides, it should be clearly stated that unless an MVP diagnosis is in fact known, the study results do not apply to other patient groups.

## Figures and Tables

**Table 1:** Patients characteristics, echocardiographic and angiographic data.

	Males (*n* = 103)	Females (*n* = 115)	*P* value
Baseline characteristics			

Age	49 ± 5	56 ± 7	NS
Arterial hypertension	7/103 (7%)	11/115 (10%)	NS
Diabetes mellitus	5/103 (5%)	7/115 (6%)	NS
Hypercholesterolemia	38/103 (37%)	46/115 (40%)	NS
Smoking	35/103 (34%)	32/115 (28%)	NS

Echocardiographic data			

Leaflet involved:			
(i) anterior	80/103 (78%)	77/115 (67%)	.048
(ii) posterior	23/103 (22%)	38/115 (33%)	.042
LVEF (%)	53 ± 2	55 ± 3	NS

Angiographic data			

Vessels involved:			
(i) 0	38/103 (37%)	52/115 (45%)	NS
(ii) 1	33/103 (32%)	37/115 (32%)	NS
(iii) 2	21/103 (20%)	18/115 (16%)	NS
(iv) 3	11/103 (11%)	8/115 (7%)	NS

LVEF: Left Ventricle Ejection Fraction, NS: Not Significant.

**Table 2:** Exercise and electrocardiographic parameters.

	Males (*n* = 103)	Females (*n* = 115)	*P* value
Exercise parameters			

Exercise duration (sec)	520 ± 32	470 ± 25	0.036
Resting systolic blood pressure (mmHg)	134 ± 12	136 ± 13	NS
Systolic blood pressure at hyperventilation (mmHg)	142 ± 11	144 ± 12	NS
Maximal systolic blood pressure (mmHg)	188 ± 16	193 ± 15	NS
Resting heart rate (bpm)	80 ± 5	78 ± 4	NS
Heart rate at hyperventilation (bpm)	106 ± 6	106 ± 5	NS
Maximal heart rate (bpm)	162 ± 9	169 ± 6	NS
Exercise-induced Angina	8/103 (8%)	6/115 (5%)	NS

Electrocardiographic parameters			

Presence of ↓ ST at *hyperventilation*:	27/103 (26%)	41/115 (36%)	.041
(i) inferior leads (II, III, aVF)	12/103 (12%)	19/115 (17%)	.045
(ii) anterior leads (V_1_, V_2_, V_3_)	0/103 (0%)	2/115 (2%)	.038
(iii) lateral leads (V_4_, V_5_, V_6_)	5/103 (5%)	7/115 (6%)	NS
(iv) inferior + lateral leads (II, III, aVF, V_4_, V_5_, V_6_)	10/103 (10%)	13/115 (11%)	NS
Maximal ↓ ST at *hyperventilation* (mV):	0.09 ± 0.03	0.11 ± 0.05	NS
(i) inferior leads (II, III, aVF)	0.09 ± 0.04	0.11 ± 0.05	NS
(ii) anterior leads (V_1_, V_2_, V_3_)	0.02 ± 0.02	0.01 ± 0.01	NS
(iii) lateral leads (V_4_, V_5_, V_6_)	0.09 ± 0.03	0.1 ± 0.01	NS
Maximal ↓ ST at *peak exercise* (mV):	0.15 ± 0.02	0.19 ± 0.03	NS
(i) inferior leads (II, III, aVF)	0.15 ± 0.05	0.19 ± 0.03	NS
(ii) anterior leads (V_1_, V_2_, V_3_)	0.04 ± 0.03	0.01 ± 0.01	NS
(ii) lateral leads (V_4_, V_5_, V_6_)	0.11 ± 0.02	0.18 ± 0.01	.041
Time for exercise-induced ↓ ST:0.1 mV (sec)	390 ± 16	362 ± 12	.043

NS: Not Significant.

**Table 3:** Correlation of ST-segment depression at hyperventilation with angiographic result.

	No-CAD (Group A, *n* = 90)	CAD (Group B, *n* = 128)	*P* value
Patients with ↓ ST at hyperventilation			

Males (*n* = 27)	21/38 (55%)	6/65 (9%)	.004
Females (*n* = 41)	33/52 (63%)	8/63 (13%)	.002
Total population (*n* = 68)	54/90 (60%)	14/128 (11%)	.001

Patients without ↓ ST at hyperventilation			

Males (*n* = 76)	17/38 (45%)	59/65 (91%)	.004
Females (*n* = 74)	19/52 (37%)	55/63 (87%)	.002
Total population (*n* = 150)	36/90 (40%)	114/128 (89%)	.001

CAD: coronary artery disease.
